# Influence of Freeze–Thaw Cycles and Sustained Load on the Durability and Bearing Capacity of Reinforced Concrete Columns

**DOI:** 10.3390/ma17246129

**Published:** 2024-12-15

**Authors:** Chen Chen, Kai Zhang, Lin Ye

**Affiliations:** 1 School of Civil Engineering, Institute of Disaster Prevention, Sanhe 065201, China; chenchen@cidp.edu.cn; 2 Key Laboratory of Building Collapse Mechanism and Disaster Prevention, China Earthquake Administration, Sanhe 065201, China; 3 Research Center of Earthquake Engineering, China Institute of Water Resources and Hydropower Research, Beijing 100048, China; 4 Ruicheng Construction Engineering Co., Ltd., Lianyungang 222042, China

**Keywords:** reinforced concrete column, aggressive environment, freeze–thaw cycles, salt erosion, sustained load, durability, bearing capacity

## Abstract

The deterioration of concrete structures is mainly due to the combined action of the environment and external load. In this study, 32 reinforced concrete columns were prepared to evaluate the coupling actions on the properties of reinforced concrete structures. The durability, bearing capacity, and failure mode of reinforced concrete columns were investigated under the combined action of freeze–thaw (F–T) cycles, sustained load, and salt corrosion (water or composite salt solution). Results show that the mass fluctuation of reinforced concrete columns under a sustained load was more obvious during F-T cycles. During the early F-T cycles, the sustained load was beneficial to the F-T resistance of the reinforced concrete columns. With the increase in F-T cycles, the damage to the columns with a sustained load gradually aggravated. In the composite salt solution, the damage to the reinforced concrete columns was postponed, and its durability showed a two-stage evolution. After 100 F-T cycles, the mass loss and relative dynamic modulus of elasticity (RDME) deterioration of the columns with a sustained load sped up significantly. The combined action of salt corrosion, load, and F-T cycles has the most significant influence on the bearing capacity, stiffness deterioration, and crack development of reinforced concrete columns.

## 1. Introduction

Concrete is the most widely used man-made construction material in the world and has been applied in hydraulic engineering, civil engineering, and other infrastructure construction [1,2,3]. In China, there is a large area of permanent and seasonal frozen soil, of which the seasonal frozen soil area accounts for 53.5% of the land area [4]. Concrete structures built in this area inevitably suffer from complex environmental effects such as frost damage, corrosion, carbonization, and salt ion erosion [5,6,7]. Frost damage has become one of the main sources of damage to concrete structures in north China, which seriously affects the service life of concrete structures [8,9,10,11].

Moreover, concrete structures are easily corroded by salt solutions, such as sulfate ions and chloride ions contained in saline soil and coastal regions, which affects the safety service and the durability of concrete structures severely [12,13,14]. In the cold region, many roads and bridges are corroded on the surface due to snow-melting agents, which leads to cracks generating, spalling, and steel bar corrosion [15,16,17,18]. However, snow melting agents are still widely used as they are inexpensive and convenient [19,20]. Current solutions to combat salt erosion in concrete include modifying cement components and refining the internal pores. These strategies usually include the use of nanomaterials, the addition of active mineral admixtures (such as SF, FA, slag powder, etc.), or the reduction in the water/binder ratio [21]. Sulphate attack represents a multifaceted process involving both physical and chemical degradation mechanisms. The physical degradation primarily stems from the crystallization of sulphates, which exerts crystallization pressure within the concrete matrix. Simultaneously, the chemical degradation arises from the reactions between sodium sulfate (Na_2_SO_4_) and cement hydration products, leading to the formation of expansive compounds such as gypsum, ettringite, and calcium silicate hydrate [22,23]. In the presence of a NaCl solution, the internal concentration gradient increases the negative pressure within concrete specimens and enhances the absorption of the solution, raising the saturation level of the specimens. This results in increased crystallization pressure and osmotic pressure, which adversely affect the anti-frost capacity of the concrete. Additionally, chloride ions (Cl^−^) in the salt solution react with cement hydration products, causing surface mortar peeling and interfacial cracking around aggregates [24,25]. As a result, it has significant meaning to study the influence of salt frost damage on the performance of concrete structures. 

Currently, the investigation of the durability of concrete materials mainly focuses on examining the influence of individual or combined factors, such as chloride, sulfate erosion, frost damage, etc. Althoey et al. [26] found that during the thermo-chemo-mechanical cycle, NaCl interacts with tri-calcium aluminate (C_3_A) and monosulfate (AFm) to cause a chemical phase transition, which caused irreversible damage to the porous cementitious materials. Rossi et al. [27] studied the relationship among the deflection, creep rate, failure rate, failure time, and load level of pre-cracking concrete. The results showed that the secondary creep deflection and the macrocrack propagation rate increase with a sustained loading level. Zhong et al. [28] analyzed the durability of pre-cracking UHPC under F-T cycles and chloride salt erosion (4 wt% NaCl solution). After 300 F-T cycles, the harmful effects of the combined action of the F-T cycles and chlorine salt erosion overcame the positive effects of the salt solution, resulting in a decline in mechanical properties. Yao et al. [29] studied the deterioration mechanism of recycled powder concrete under the coupling effect of 5% sulfate solution and freeze–thaw cycle and concluded that, with the increase in the number of freeze–thaw cycles, the physical and mechanical properties of RPC deteriorated, the critical pore size increased, and the corrosive products ettringite and gypsum were formed. Gou et al. [30] studied the evolution of the mechanical properties and durability of new lightweight, high-strength engineering cementing composites under the triple coupling effects of chloride attack, sulfate attack, and a freeze–thaw cycle. It is indicated that the combined physical and chemical action of sulfate promotes the diffusion and penetration of chloride ions, while chloride ions delay the diffusion and expansion reaction of sulfate. Therefore, it is particularly important to gain insight into the underlying mechanisms behind specific destructive behaviors in these aggressive environments.

The coupling action of sustained load and environment has significant effects on the creep, corrosion, mechanical properties, and durability of reinforced concrete columns. Bao et al. [31] studied the water permeability characteristics of concrete under a sustained load and F-T cycles. The results show that stress ratio and frost damage have significant effects on the capillary water absorption and permeability of concrete. The initial capillary absorption coefficient and water permeability coefficient increase with the increase in F-T cycles and reduce first and then increase with the increase in sustained load. Li et al. [32] studied the effects of load and reinforcement corrosion on the mechanical properties of concrete columns. The results show that the coupling of reinforcement corrosion and sustained load significantly aggravates the degradation of reinforced concrete columns, and the failure mode changes to brittle failure. Mehta [33] and Valenza et al. [34] considered that the important factors affecting the durability of concrete structures and materials are as follows: reinforcement corrosion > frost damage > ion chemical erosion. Bao et al. [35] studied the effect of a sustained load on the capillary water absorption of recycled concrete aggregates and analyzed the quantitative relationship between adsorption rate and stress level.

The existing research results show that continuous complex stress conditions and environmental coupling can induce the expansion of initial microcracks in concrete and the accumulation of internal damage and a decline in the macroscopic mechanical properties of the material. In this condition, it is no longer suitable to accurately evaluate the structural resistance under a long-term load by relying solely on the material performance parameters obtained from the static test in the laboratory. At present, the prediction of the durability of a concrete structure is mostly based on the non-damage assumption and the law obtained by laboratory testing, which may overestimate the durability and reliability of a concrete structure. Moreover, studies on the effects of sustained loads and salt corrosion on the degradation of reinforced concrete under F-T cycles are not comprehensive. Therefore, it is necessary to carry out research on the influence of sustained loads, salt corrosion, and F-T cycles on the mechanical properties and durability of reinforced concrete columns, which can provide an important supplement for evaluating the safety of concrete structures in service.

In this work, a series of experiments were carried out on reinforced concrete columns under the combined action of F-T cycles, composite salt solution erosion, and a sustained load. The coupling action of environment and load on the durability, bearing capacity, and failure mode of reinforced concrete columns was systematically investigated. The test results can offer references for the design and evaluation of reinforced concrete column durability in aggressive environments. 

## 2. Materials and Methods

### 2.1. Material and Mixture

In the test, the selected cement was 42.5 ordinary Portland cement. The chemical composition and physical properties are listed in Table 1, according to Chinese specification GB 175-2023 [36], which is similar to ASTM C 1084-2002 [37]. Gravel with an apparent density of 2680 kg/m^3^ and particle size of 5~25 mm was selected as the coarse aggregate. River sand with a fineness modulus of 2.7 was used. The mixing water was ordinary tap water.

Concrete columns were prepared and fabricated in the laboratory according to the design method of Chinese specification JGJ 55-2011 [38]. The mix proportions used in this test are shown in Table 2. The measured slump was 100 mm. After 7 days and 28 days of standard curing (temperature was 20 ± 3 °C; relative humidity was 95% RH), the tested compressive strength of the 150 mm cubic specimen was 22 MPa and 33.2 MPa, respectively.

**Figure 1:**
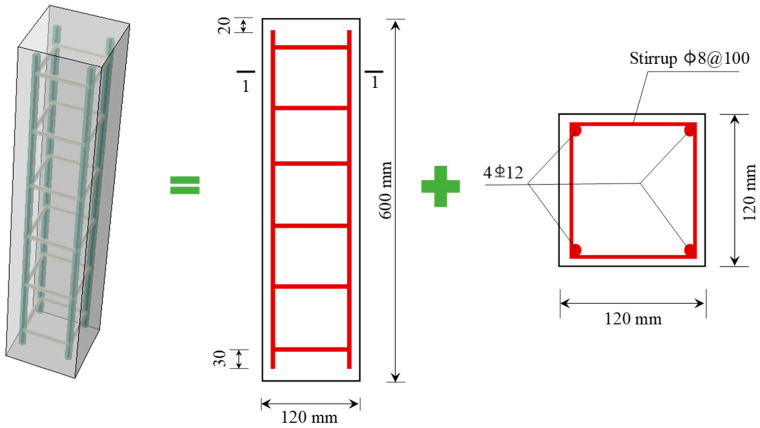
Schematic diagram of reinforced concrete column.

A total of 32 reinforced concrete columns were prepared, and the size was 120 mm × 120 mm × 600 mm. A diagram of the reinforced concrete column is shown in Figure 1. The stirrups were steel bars with a diameter of 8 mm and a spacing of 100 mm. The longitudinal bars were Grade II steel with a diameter of 12 mm.

### 2.2. Experiment Methods

#### 2.2.1. Design of Load Cases

In order to study the durability and bearing capacity deterioration of reinforced concrete in an aggressive environment, the influence factors of sustained load, composite salt corrosion, and F-T cycles were selected in this test. The load cases considered were as follows: (1) the individual action of F-T cycles; (2) the combined action of a sustained load and F-T cycles; (3) the combined action of salt corrosion and F-T cycles; (4) the combined action of a sustained load, salt corrosion, and F-T cycles. The specific experimental design conditions are listed in Table 3.

#### 2.2.2. Sustained Loading Device

**Figure 2:**
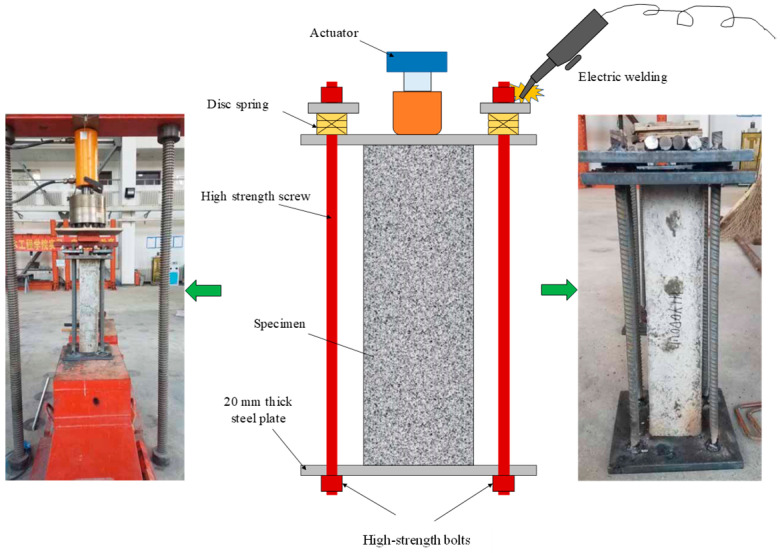
Diagram of sustained loading device.

The testing device for sustained loading on a reinforced concrete column is illustrated in Figure 2. This sustained loading device is designed based on the principle of post-tensioning pre-stressing. The load on the columns, which is 70% of the predetermined peak load, is applied and maintained by a hydraulic actor. Then, high strength bolts and electric welding are employed to fix the 20 mm thick steel plates to each end of the column, which ensures the specimen is under sustained loading.

In this experiment, the selected disc spring is Type A, measuring 56 mm in diameter, 28.5 mm in height, and 3 mm in thickness. The spring has an elastic modulus E = 206 GPa and a Poisson’s ratio μ = 0.3. When the spring reaches 75% of its maximum deformation, the load is 11,400 N. A reinforced concrete column is fixed by four high-strength screws, and each high-strength screw is equipped with five disc springs. The load applied to the disc springs is 210 kN, which ensures the deformation reserve of the device and prevents stress relaxation caused by creep.

#### 2.2.3. Composite Salt Solution

The concentration of the composite salt solution utilized in this study is, according to the soil samples collected from 30 cm under the ground surface of Da’an City, Jilin Province, where there is one of the largest saline soil distribution areas in China. The composition and mass fraction of the composite salt solution are listed in Table 4. The methodology of the F-T cycles employed in this test is based on the Chinese national standard GB/T 50082-2009 [39] and ASTM C666/C666M-15 [40] and ASTM C 1262a-05 [41]. During the experimental procedure, the mass change of the columns and the ultrasonic pulse velocity (UPV) were measured after every 25 F-T cycles. Furthermore, the maximum number of F-T cycles was 150 to comprehensively evaluate the durability and corrosion resistance of the reinforced concrete columns under F-T and salt erosion environments.

#### 2.2.4. Salt Solution Corrosion

**Figure 3:**
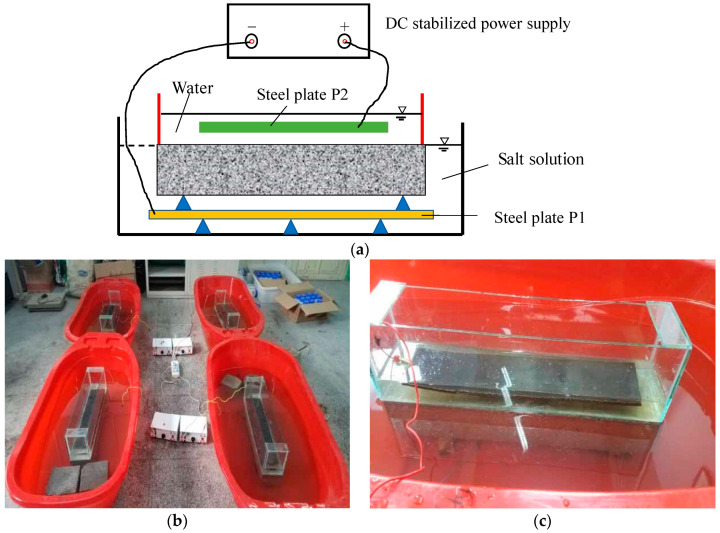
Schematic diagram of electromigration-accelerated ion corrosion device. (**a**) Working principle, (**b**) full view of the test, (**c**) local view of the test.

According to ASTM C 1262-05 [41] and reference [42], a salt corrosion test scheme was designed to accelerate the indoor salt corrosion test without causing deterioration different from the natural environment by using a constant current acceleration method. The salt corrosion includes two stages: accelerated migration corrosion (Stage I) and immersion corrosion (Stage II). The schematic diagram of electromigration-accelerated ion corrosion is shown in Figure 3. A glass container with tap water was placed on the top surface of the concrete column and was bonded to the top surface with structural adhesive to ensure sealing. The entire column was immersed in the prepared composite salt corrosion solution, and two stainless steel plates were placed at the bottom of the composite salt solution and the water, respectively, which are marked as P1 and P2. A direct current (DC) stabilized power source of 14 V was applied between P1 and P2. P1 was connected to the negative electrode, and P2 was connected to the positive electrode. The electromigration acceleration lasted 7 days, and the change in mass and dynamic modulus was recorded every day. The change in mass and UPV were recorded every 5 days during the 30 days of immersion corrosion. To avoid water evaporation and electromigration consumption, each column was reconfigured with new composite salt solutions before immersion corrosion to ensure the salt solution concentration was consistent with the designed concentration.

#### 2.2.5. Axial Loading Test

**Figure 4:**
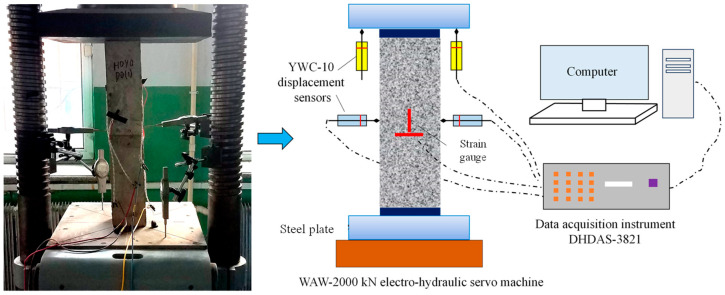
Schematic of axial loading device.

Following exposure to varying numbers of F-T cycles, axial compression bearing capacity tests were performed on the reinforced concrete columns. Figure 4 shows the schematic diagram of the axial compression strength test device. Considering that the surface of the column becomes rough after experiencing F-T cycles and salt corrosion, the end of the column was first polished. Subsequently, displacement sensors were installed to measure the deflection, and the dynamic signal acquisition instrument was connected to collect the measured data. Then, the specimens were placed at the geometric center of the upper and lower loading plates and preloaded three times to mitigate the effects of uneven ends and gaps between these plates. The preloading was 5% of the estimated ultimate bearing capacity. After that, the column was stage loaded. For the first stage, the load increment was 30 kN with a loading rate of 2 kN/s until reaching 80% of the estimated capacity. For the second stage, the load increment was 20 kN with a loading rate of 1 kN/s until column failure. At the end of each load increment, the load was held for 15 min. 

## 3. Results and Discussion

### 3.1. Failure Characteristics After Frost Damage

**Figure 5:**
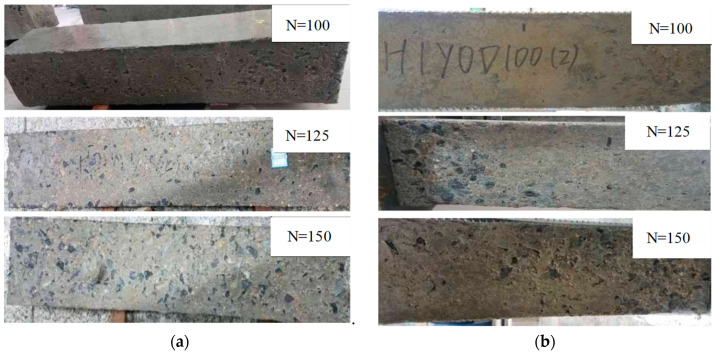
Damage after different F-T cycles under water medium. (**a**) H0Y0, (**b**) H1Y0.

The frost damage to the columns after the F-T cycles is illustrated in Figure 5. It can be seen that, before 100 F-T cycles, the surface of the unloaded reinforced concrete column shows basically no significant change. After 125 F-T cycles, the surface mortar of the column spalls off, and a small amount of coarse aggregate is exposed near the end of the column. At the end of 150 F-T cycles, the surface mortar of the column spalls off seriously, and more coarse aggregates are exposed. Moreover, the end of the column is obviously damaged, which is consistent with the conclusions of references [8,23,26].

**Figure 6:**
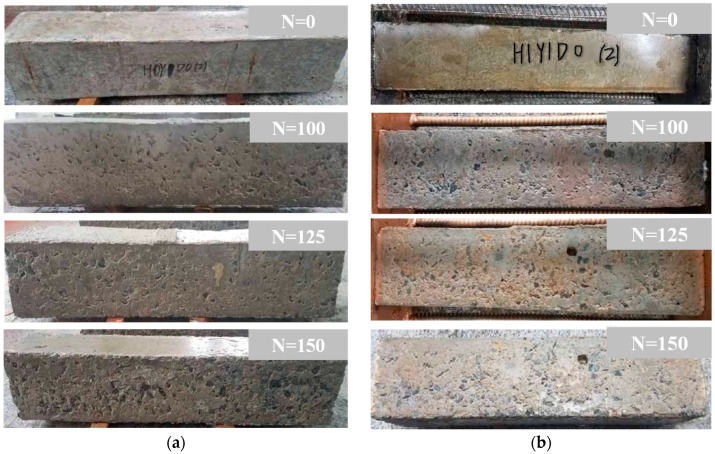
Damage after F-T cycles under composite salt solution. (**a**) H0Y1, (**b**) H1Y1.

Figure 6 demonstrates the damage of the reinforced concrete column after different F-T cycles under a composite salt solution. Figure 6a represents the deterioration of the unloaded specimens. Due to electrochemical ion corrosion, the surface of the reinforced concrete column connected to the positive electrode shows obvious rusting. After 75 F-T cycles, the surface mortar starts to spall, and the surfaces of the column become loose after 100 F-T cycles. After 125 F-T cycles, the coarse aggregate is exposed and even falls at the corner of the column. After 150 F-T cycles, the deterioration of the reinforced concrete columns is aggravated. Compared with Figure 5a, it can be seen that the surface deterioration of the reinforced concrete column is more serious under the combined action of salt solution erosion and F-T cycles.

Figure 6b shows the apparent failure characteristics of the sustained loaded columns under the combined action of salt solution erosion and F-T cycles. It can be noticed that after 100 F-T cycles, the surfaces of the columns have a large area of holes and become rough. After 150 F-T cycles, a large area of mortar falls, and many coarse aggregates are exposed.

### 3.2. Mass Loss Rate

**Figure 7:**
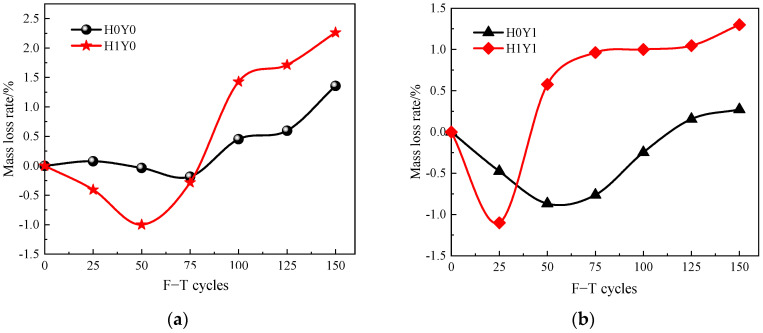
Mass loss rate after frost damage. (**a**) H0Y0 and H1Y0, (**b**) H0Y1 and H1Y1.

Figure 7 shows the relationship between the mass loss rate of the reinforced concrete columns and the number of F-T cycles. Figure 7a,b shows the results in water and in the salt solution, respectively. From Figure 7a, it can be seen that the mass changes of the loaded column are more significant than that of the unloaded column. The mass of the loaded reinforced concrete column increases slightly at first and then decreases gradually with the increase in F-T cycles. After 50 F-T cycles, the mass loss rate of the loaded column is −1.0%. Then, the mass declines rapidly. After 150 F-T cycles, the mass loss rate of the loaded column reaches 2.26%.

From Figure 7b, it can be seen that, in the salt solution, the mass of both the loaded and unloaded columns increases first and then decreases. For the unloaded column in salt solution, the mass increases before 75 F-T cycles. After that, the deterioration of the unloaded column aggravates, and the mass starts to decrease. After 150 F-T cycles, the mass loss rate reaches 1.4%. For the loaded column in salt solution, the mass loss rate increases after 50 F-T cycles, which is more obvious than that of the unloaded column in the salt solution. After 150 F-T cycles, the mass loss rate reaches 2.2%. This is because there are microcracks generating in the loaded reinforced concrete column. At the early stage of the F-T cycles, the microcracks gradually saturate by absorbing water through capillary action, resulting in the mass increasing. With the increase in F-T cycles, the microcracks develop and expand gradually under the action of periodic frost-heaving stress [8,20,22]. This results in the cracking and spalling of the surface mortar and eventually leads to the mass of column decline.

### 3.3. RDME After Frost Damage

**Figure 8:**
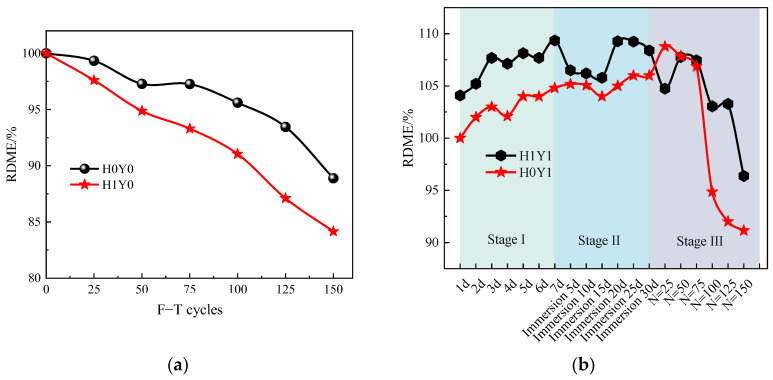
RDME under F-T cycles. (**a**) H0Y0 and H1Y0, (**b**) H0Y1 and H1Y1.

Figure 8 shows the RDME changes of reinforced concrete columns under different load cases after the F-T cycles. Figure 8a is the RDME of the column in water. It can be seen that, for both loaded and unloaded specimens, the RDME of the reinforced concrete columns decreases with the increase in F-T cycles. Especially, the RDME of H1Y0 decreases faster than that of H0Y0, which means the sustained load increases the frost damage of the reinforced concrete column.

The RDME variation of the reinforced concrete specimens under a composite salt solution is shown in Figure 8b. Stage I represents the process of electrochemical-accelerated ion migration, stage II is the change in the RDME after the full immersion in the composite salt solution, and stage III represents the change in the RDME after F-T cycles under a composite salt solution. From Figure 8b, it can be seen that before the F-T cycles, the RDME of both H1Y1 and H0Y1 shows a fluctuation-increasing trend. This is because during the process of salt solution erosion, physical and chemical reactions occur between the hydration products inside the concrete and salt solution, producing ettringite, gypsum, calcite, and other products [37]. This improves the compactness of the concrete and is beneficial to the anti-frost performance. After 100 F-T cycles, the RDME of H0Y1 decreases rapidly and then slows down. After 150 F-T cycles, the RDME of H0Y1 decreases to 91.14%. 

For H1Y1, the RDME decreases significantly after 125 F-T cycles, and the RDME reduces to 96.37% after 150 F-T cycles. This is mainly due to the fact that, with the increase in F-T cycles, the crystallization expansion pressure is formed as the salt solution precipitates crystalline products [13,14,17]. At the same time, due to the difference in internal and external concentration, osmotic pressure and internal stress are generated, resulting in the generation, expansion, and penetration of internal microcracks. Moreover, the salt solution erosion reduces the pH value of the concrete [20], which accelerates the corrosion and rust expansion of the steel bars and causes the concrete to crack. Macroscopically, the RDME decreases.

### 3.4. Failure Mode

**Figure 9:**
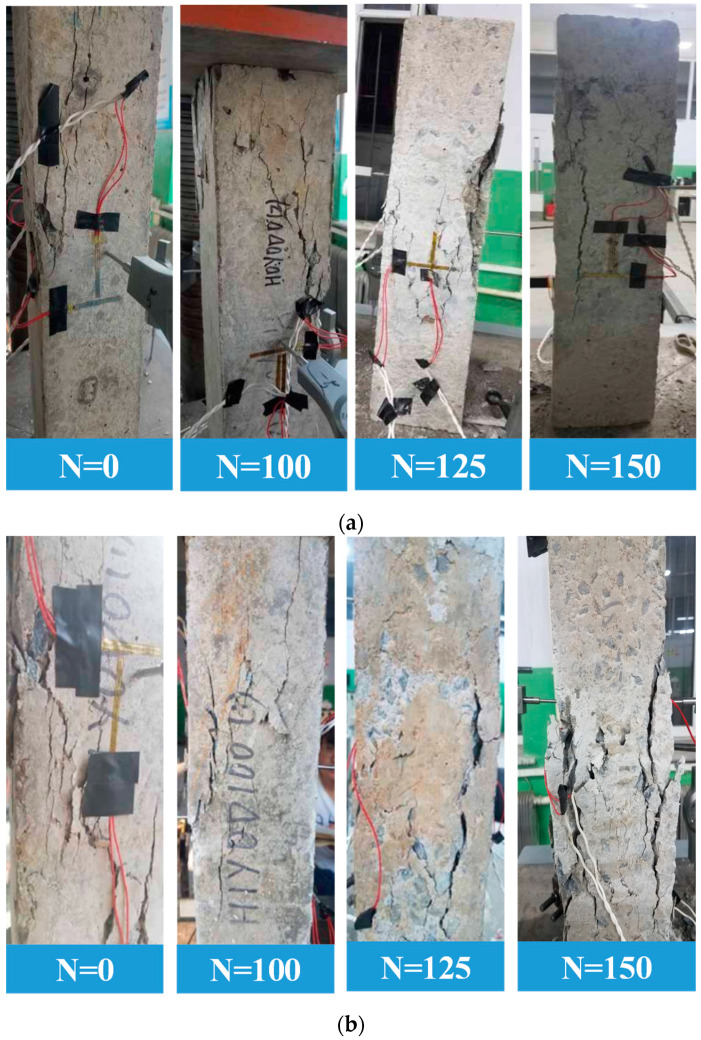

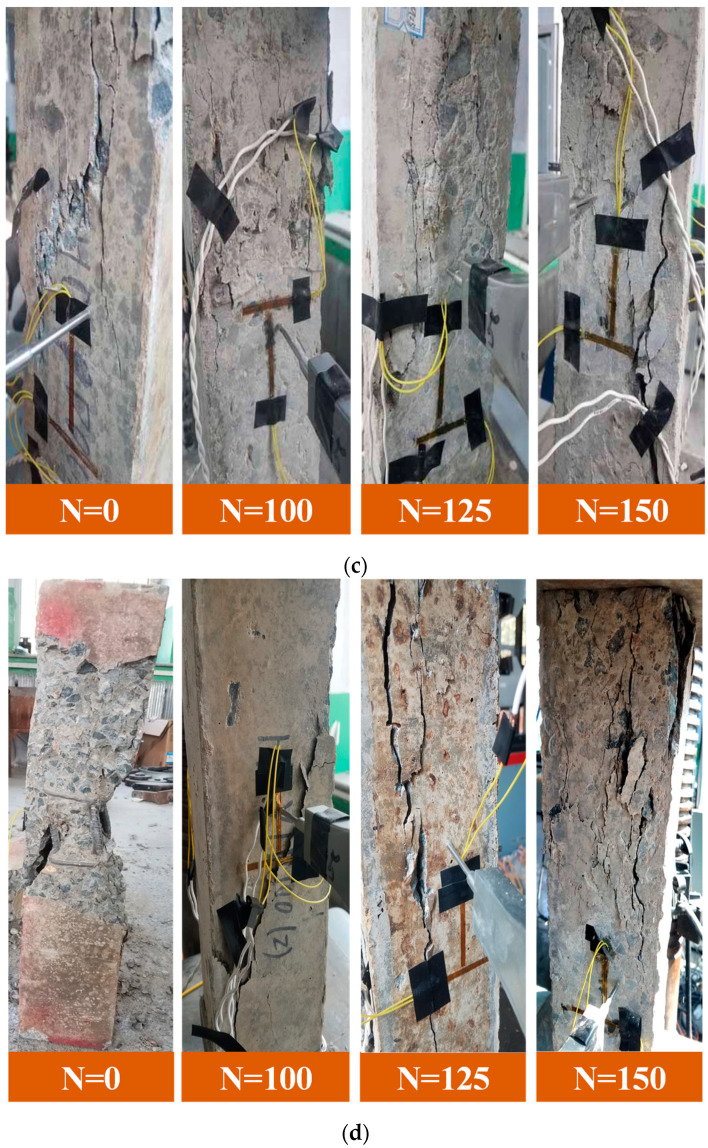
Failure characteristics of reinforced concrete columns under axial loading with different F-T cycles. (**a**) H0Y0, (**b**) H1Y0, (**c**) H0Y1, (**d**) H1Y1.

Figure 9 shows the failure modes of the reinforced concrete columns under axial loading after different F-T cycles. It can be seen that there is no significant difference between the final failure modes of H0Y0, H1Y0, H0Y1, and H1Y1. Before the F-T cycles, the compactness of the column was high, and the failure mode was basically brittle failure with only a few vertical cracks on the surface. With the increase in F-T cycles, the inner compactness of the column decreases, the surface of the specimen becomes rougher, and the number of microcracks on the surface gradually increases. After 150 F-T cycles, the columns demonstrate obvious plastic or ductile failure, and the cracks gradually extend and expand from the initial microcracks parallel to the loading direction into wider cracks. Finally, the diagonal shear failure mode of the columns is formed.

**Figure 10:**
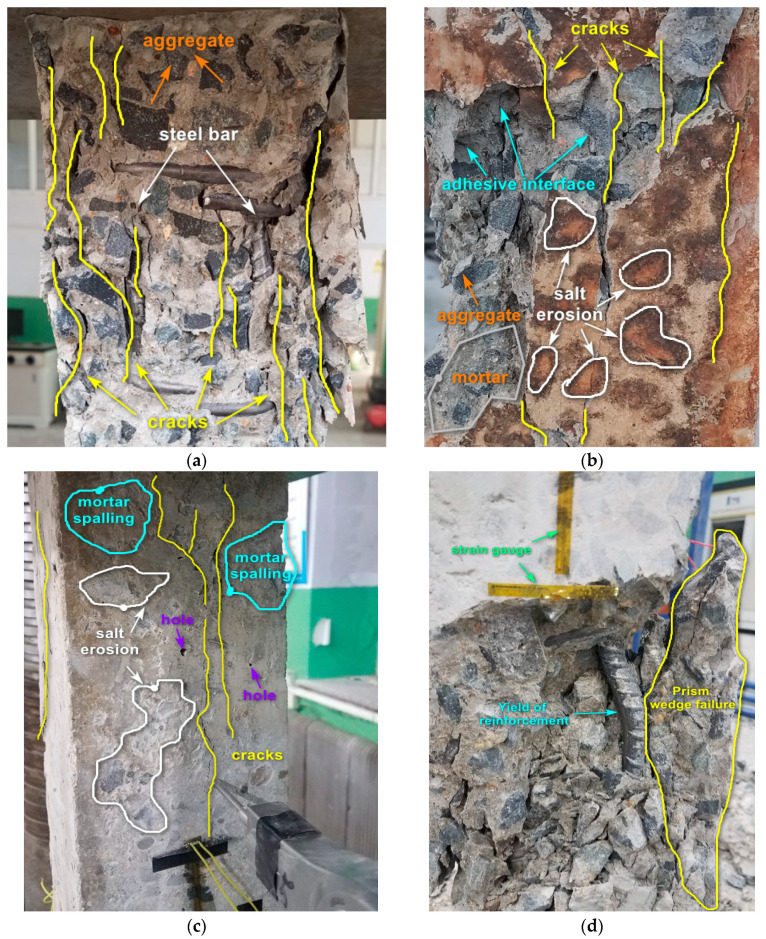
Local failure characteristics of reinforced concrete columns under axial compression with different F-T cycles. (**a**) H0Y0D0, (**b**) H1Y1D125, (**c**) H0Y1D125, (**d**) H1Y0D150.

The local failure characteristics of each group of reinforced concrete columns are illustrated in Figure 10. It can be observed from Figure 10a that the failure surface of the group H0Y0D0 sample exhibits relatively uniform behavior, with cracks primarily propagating along the bonding interface between aggregates and mortar. Several vertical cracks parallel to the loading direction are present, accompanied by minimal aggregate crushing. The longitudinal reinforcement shows yielding. Ultimately, transverse swelling and failure occur at the center of the reinforced concrete column. Figure 10b depicts the localized failure characteristics of the reinforced concrete column H1Y1D125 subjected to the combined effects of sustained loading, salt corrosion, and F-T cycles. The specimen surface displays significant rust traces, and cracks also primarily propagate along the bond interface between aggregates and mortar. In Figure 10c, a reinforced concrete specimen exhibits localized failure after undergoing 125 F-T cycles under salt corrosion conditions. The specimen surface shows evidence of salt corrosion with multiple areas of mortar spalling and exposed aggregates. Two primary cracks are observed on the surface parallel to the loading direction, gradually extending from top to bottom. Additionally, multiple voids are present on the specimen surface. Figure 10d depicts the failure characteristics in the reinforced concrete column H1Y0 under sustained loading after undergoing 150 F-T cycles. Internal reinforcement undergoes bending deformation leading to outward bulging and ultimately causing surrounding concrete failure due to load compression.

In the early stages, the presence of the salt solution lowers the freezing point, temporarily mitigating frost damage [14,17,42]. However, in a NaCl solution, calcium hydroxide (Ca(OH)_2_) precipitates, and microcracks form on the concrete surface [43]. The mismatch in physical properties between aggregates and cement mortar, coupled with cyclic temperature fluctuations, induces expansive cracking within the concrete. This phenomenon exacerbates frost damage, leading to the progressive deterioration of concrete cores.

F-T cycles progressively degrade the impermeability of concrete, creating additional pathways for sulfate and chloride ions to penetrate the concrete matrix. Sulfate attack exacerbates this process by causing initial microcracks to expand into microfractures, further accelerating the ingress of salt ions. This synergistic interaction between frost damage and chemical erosion significantly intensifies the deterioration of the concrete structure [44,45].

### 3.5. Bearing Capacity

**Figure 11:**
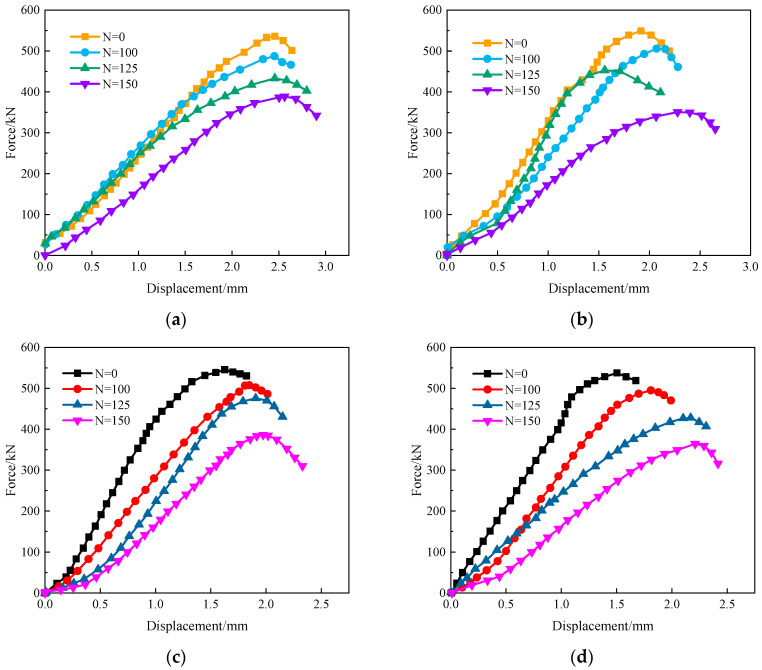
Force–displacement curves of reinforced concrete columns under axial compression with different F-T cycles. (**a**) H0Y0, (**b**) H1Y0, (**c**) H0Y1, (**d**) H1Y1.

Figure 11 shows the load–displacement curves of the reinforced concrete columns under different F-T cycles. It can be seen that the load–displacement curves of the columns are similar. With the increase in the F-T cycles, the peak load of the reinforced concrete columns in the same load case gradually decreases and shifts to the right. The upward part of the curve gradually becomes more and more concave. This is due to the gradual generation and expansion of microcracks in the concrete during the process of the F-T cycles and the continuous evolution of internal damage in the concrete. At the initial stage of loading, the microcracks are first compacted and gradually closed, which is reflected in the nonlinear change characteristic of the downward concave shape of the loading curve, which coincides with the results from references [22,23]. 

Furthermore, after 100 F-T cycles, the decline rate of the bearing capacity of the sustained loaded reinforced concrete columns is obviously accelerated. After 150 F-T cycles, the bearing capacity of H0Y0 and H1Y0 decreases by 27.5% and 36.2%, respectively. For the columns after the treatment with a composite salt solution, the bearing capacity of the sustained-loaded columns and unloaded columns reduces by 29.2% and 32.2%, respectively. Overall, the bearing capacity of H1Y1 is lower than that of H0Y1. Besides, the composite salt solution accelerates the corrosion of the internal steel bars. 

**Figure 12:**
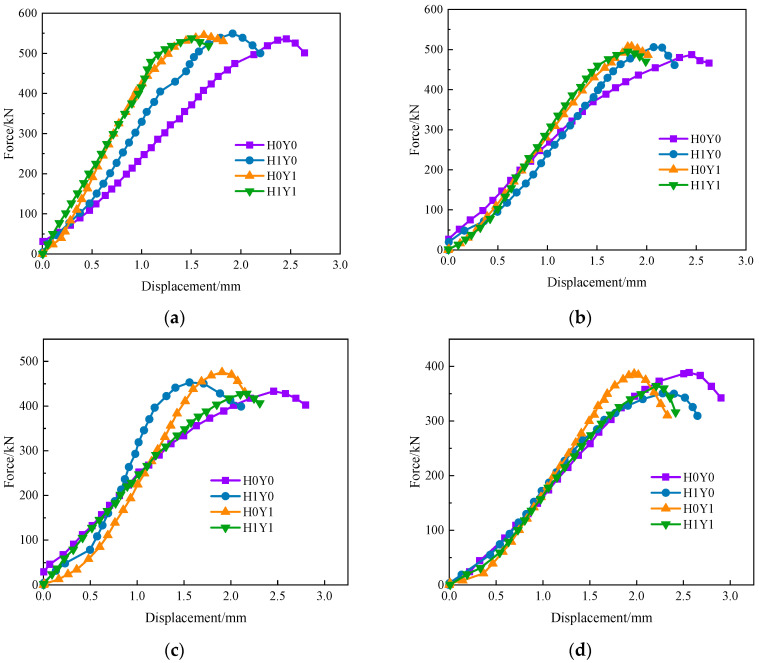
Force–displacement curves of reinforced concrete columns under different conditions with the same F-T cycles. (**a**) N = 0 cycles, (**b**) N = 100 cycles, (**c**) N = 125 cycles, (**d**) N = 150 cycles.

Figure 12 shows the force–displacement curve of the concrete columns under different load cases with the same F-T cycles. It can be seen that, after sustained loading and salt corrosion treatment, the maximum value of the force–displacement curve of H0Y0 moves clearly to the right before the F-T cycles. This is because of the increase in concrete compactness and strength, which is represented by the ascending segment of the force–displacement curve shifting to the left. From Figure 8b, it is noticed that the elastic modulus of the reinforced concrete columns treated with salt solution fluctuates and increases, which is basically consistent with the conclusions in reference [46]. With the increased number of F-T cycles, the difference in the load–displacement curves obtained by each group gradually decreases, the rising section of the curve gradually becomes flat, the peak stress decreases, and the stiffness decreases. The load–displacement curves of the other three groups show a more obvious trend to the right, except H0Y0. This means that, under the same F-T cycles, both sustained loading and salt corrosion have significant effects on the deformation capacity of reinforced concrete columns, and the effect of sustained loading on the bearing capacity of reinforced concrete is higher than that of salt corrosion.

### 3.6. Stress–Strain Curve

**Figure 13:**
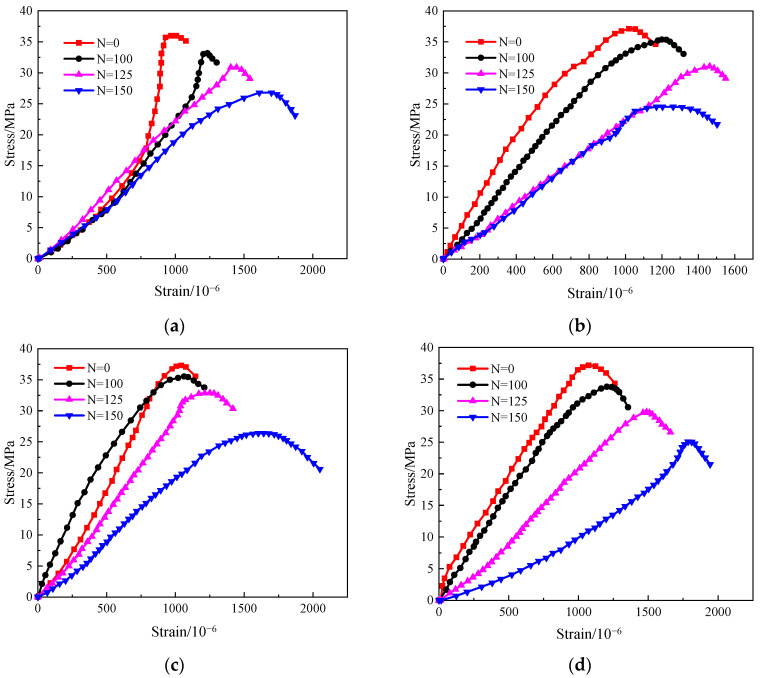
Stress–strain curves of reinforced concrete columns under axial compression with different F-T cycles. (**a**) H0Y0, (**b**) H1Y0, (**c**) H0Y1, (**d**) H1Y1.

Figure 13 presents the stress–strain curves after various numbers of freeze–thaw cycles (N = 0, 100, 125, and 150 cycles). As observed from the figure, the stress–strain curves of the reinforced concrete columns exhibit similar trends under all tested conditions. With an increasing number of freeze–thaw cycles, the slope of the ascending segment of the curves decreases, indicating a reduction in the stiffness of the reinforced concrete columns. The peak stress gradually diminishes as the number of freeze–thaw cycles increases, while the peak strain consistently increases. This phenomenon is attributed to alterations in the micro-pore structure within the reinforced concrete columns resulting from the combined effects of freeze–thaw cycling, salt corrosion, and sustained loading. These alterations cause gradual interconnection among some pores, leading to a redistribution of internal forces and the generation of damage from surface to interior. On one hand, this results in a decline in mechanical properties; on the other hand, it leads to a compaction effect within the reinforced concrete, manifesting as decreased stiffness, increased strain, and an enhanced plastic deformation capacity. These findings are consistent with conclusions drawn in previous studies [12,30,47].

### 3.7. Compressive Strength Decay Models

**Figure 14:**
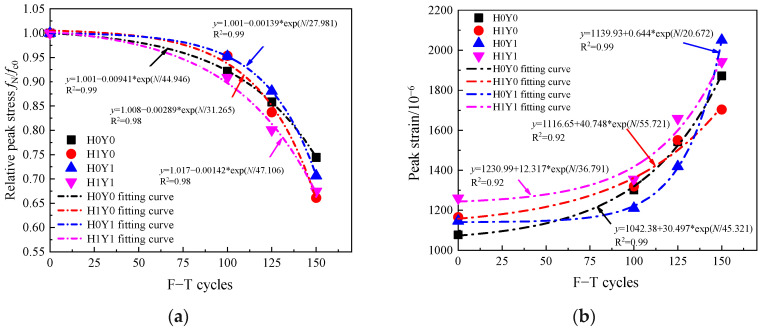
Stress–strain curves of reinforced concrete columns under axial compression with different F-T cycles. (**a**) relative peak stress, (**b**) Peak strain.

According to the experimental results, Figure 14a,b illustrates the variations of relative peak stress and peak strain, respectively, with the number of F-T cycles under different load cases. The figure demonstrates a consistent decrease in relative peak stress as the number of freeze–thaw cycles increases, while peak strain exhibits an opposite trend of continuous augmentation. It is worth noting that minimal changes are observed in peak strain before 100 F-T cycles. However, a significant acceleration in the rate of peak strain growth becomes evident after 125 cycles. An exponential function decay model can effectively depict the relationship between both relative peak stress and peak strain with respect to the number of F-T cycles; this is consistent with the phenomenon observed by Bao et al. [22].

## 4. Further Discussion

Sulphate attack represents a multifaceted process involving both physical and chemical degradation mechanisms [21,29]. The physical degradation primarily stems from the crystallization of sulphates, which exerts crystallization pressure within the concrete matrix [22,23,26]. Concurrently, the chemical degradation arises from the reactions between sodium sulfate (Na_2_SO_4_) and cement hydration products, leading to the formation of expansive compounds such as gypsum, ettringite, and calcium silicate hydrate [30,43]. The generation of these expansive materials induces internal stresses within the concrete. The synergistic interaction between these physical and chemical mechanisms ultimately results in the deterioration of concrete structures.

The damage inflicted by sodium sulfate on concrete involves three interconnected processes: the diffusion of sulfate ions, chemical reactions between sulfates and cement hydration products, and the crystallization of corrosion products (ettringite and gypsum) [20,25]. Sulfate ions diffuse into the concrete matrix and initially react with calcium hydroxide to form gypsum. Due to the low activation energy of the reaction leading to ettringite formation, the gypsum subsequently reacts with hydrated aluminates or incompletely hydrated C_3_A to produce ettringite [26,34].

The influence of a composite salt solution, sustained loading, and the F-T cycles on the reinforced concrete columns is complex, having both positive and negative damage effects [14,17,20]. On one hand, the sulfate and chloride reduce the freezing point of pore water in the concrete, increase the compressibility of the ice, and alleviate the combined damage of sulfate and chloride after the F-T cycles [28,29]. On the other hand, the F-T failure results in microcracks generating in the concrete, which accelerates ion invasion. Meanwhile, the initial saturation degree of concrete is increased by the sulfate and chloride composite solution [18,34]. The supersaturated crystallization of the composite salt solution increases pressure in the capillary, and the crystallized salt aggravates the frost damage. The process of a chemical attack of sulfate on concrete is mainly that sulfate ions infiltrate into concrete and react with calcium hydroxide in the cement, forming gypsum [9,30,48]. 

In the presence of a NaCl solution, the internal concentration gradient increases the negative pressure within concrete specimens and enhances the absorption of the solution, raising the saturation level of the specimens. This results in increased crystallization pressure and osmotic pressure, which adversely affect the anti-frost ability of the concrete. Additionally, the chloride ions (Cl^−^) in the salt solution react with the cement hydration products, causing surface mortar peeling and interfacial cracking around the aggregates. In addition, the sulfate ions react with the hydrated aluminum phase and aluminum-containing gel to form ettringite, resulting in the solid phase volume increasing by 94%. Moreover, the expansion rate of the NaCl solution increases after freezing, which is adverse to the anti-freezing performance of concrete [16,17,20]. 

## 5. Limitations of Research

(1) The durability of a concrete structure and its evaluation method are scientific and technical problems that have drawn increasing attention. Previous studies only focused on the mechanical properties and durability under the action of a single environmental factor, which is obviously inconsistent with the real service environment. Therefore, it is necessary to shift from considering individual environmental factors to simultaneously considering the interaction of mechanical, environmental, and material factors. However, it often takes a lot of manpower, material resources, and monitoring equipment to carry out tests under real environmental conditions, and it also takes a long time.
(2) In this work, the effect of F-T cycles and load on concrete column strength and crack generation is studied, which is immediate and significant. However, for the long-term durability of concrete structures, it is more critical to understand how these effects accumulate over time and ultimately lead to a decline in the performance of the concrete.

Therefore, in order to understand the durability of concrete structures comprehensively, the long-term effects of loads, especially the cumulative effects of loads combined with F-T cycles, need to be further discussed in future studies. Additionally, considering that the actual environment of concrete structures is usually more complex than in laboratory conditions, future research should also consider more practical factors, such as different climatic conditions, different concrete types and mix ratios, and different load types, as well as their action mode. These factors significantly affect the durability of the concrete and need to be fully considered in experimental designs.

(3) The performance evolution of concrete structures in the service environment is a long and complicated process, and it is difficult to reproduce the real service environment in laboratory tests. The differences exist not only in the material properties but also in uncertainties about the location of the exposure environment and the diversity of loading conditions. Moreover, the traditional test methods often cause damage to the concrete structure on site and cannot directly obtain the structural state under the real service environment.

In this experiment, 32 reinforced concrete columns were prepared with the concrete mix ratio commonly used in practical engineering. After accelerating ion erosion through short-term indoor electrification, the performance deterioration tests of reinforced concrete columns under the action of sustained loading and F-T cycles were carried out. The deterioration law of bearing capacity after salt solution erosion and F-T cycles is emphasized, but the influence of exposure position and real stress state in the actual environment is not considered. Although there are some limitations in the laboratory test and the test results may not accurately reflect the performance of the actual structure, the change trend in the mechanical properties and the durability of reinforced concrete columns under the action of sustained load, salt ion erosion, and F-T cycles has been verified and perfected. Therefore, it is necessary to study the evolution of concrete properties under the action of multi-factor coupling, which can provide more reliable support for the design, construction, and maintenance of concrete structures.

## 6. Conclusions

In order to study the durability, bearing capacity, and failure mode of reinforced concrete columns under the combined action of F-T cycles, composite salt solution erosion, and a sustained load, a series of experiments were carried out in this study. The following conclusions can be drawn:

(1) Under the coupling action of composite salt solution corrosion, sustained loading, and F-T cycles, the bearing capacity, stiffness, and durability of reinforced concrete columns reduce significantly. The final deterioration evolution is a progressive and irreversible complex failure process with the superimposed influence of physical and chemical erosion.
(2) Under the combined action of sustained loading and F-T cycles, the mass loss rate and the deterioration degree of the RDME of the reinforced concrete column gradually increase. Moreover, both the surface scaling and the internal damage of the reinforced concrete columns become more severe.
(3) Under the combined action of a salt solution, a sustained load, and F-T cycles, the RDME shows a trend of decreasing fluctuations. After 150 F-T cycles, the RDME of H0Y0, H1Y0, H0Y1, and H1Y1 reduces by 88.8%, 84.2%, 91.1%, and 96.3%, respectively, which indicates that the F-T failure of the reinforced concrete columns is aggravated by sustained loading.
(4) The trend of load–displacement curve changes little under the combined action of a composite salt solution, a F-T environment, and a sustained load. With the increase in F-T cycles, the peak load of reinforced concrete columns gradually decreases and shifts to the right, and the slope of the rising section becomes increasingly flat under the same load case. After 150 F-T cycles, the bearing capacity of H0Y0, H1Y0, H0Y1, and H1Y1 decreases by 27.5%, 36.2%, 29.2%, and 32.2%, respectively. Although there is little numerical difference, the trend of bearing capacity reduction in H1Y0 and H1Y1 under the F-T cycles accelerates gradually.
(5) It can be seen from the final failure characteristics that the presence of a composite salt solution accelerates the corrosion of steel bars inside the reinforced concrete column, which shows multiple coupling effects. Overall, the ultimate bearing capacity is affected in the following way: H1Y1 > H0Y1 > H1Y0 > H0Y0.

Furthermore, this work needs to carry out research on the evolution of concrete mechanical properties and durability under the combined action of various salt solution concentrations, different composite salt solution concentrations, different erosion environments, and F-T cycles, which will be the focus of future research.

## Figures and Tables

**Table 1:** Chemical composition and physical properties of cement.

Chemical Components	Content (%)
SiO_2_	20.91
CaO	61.75
Al_2_O_3_	5.58
Fe_2_O_3_	3.04
MgO	2.07
NaO	0.8
SO_3_	3.11
LOI	2.75
Specific surface area/(m^2^/kg)	330
Density/(kg/m^3^)	3100
Initial setting time/min	155
Final setting time/min	215

**Table 2:** Mix proportions used for concrete specimens (kg/m^3^).

Type	w/c	Cement	Water	Coarse Aggregate	Fine Aggregate	Water-Reducing Agent
C30	0.478	324	178.2	1222	658	2.59

**Table 3:** Details of experimental scheme.

Group	Sustained Load	Composite Solution	F-T Cycles	Group	Sustained Load	Composite Solution	F-T Cycles
H0Y0D0	0	0	0	H0Y1D0	0	1	0
H0Y0D100	0	0	100	H0Y1D100	0	1	100
H0Y0D125	0	0	125	H0Y1D125	0	1	125
H0Y0D150	0	0	150	H0Y1D150	0	1	150
H1Y0D0	1	0	0	H1Y1D0	1	1	0
H1Y0D100	1	0	100	H1Y1D100	1	1	100
H1Y0D125	1	0	125	H1Y1D125	1	1	125
H1Y0D150	1	0	150	H1Y1D150	1	1	150

Note: H0 indicates without sustained load; Y0 means that the F-T medium is water; and D100 represents 100 rounds of F-T cycles.

**Table 4:** Mass fraction of composite salt solution and the dosage of various salts.

Type	Salt Type and Dosage/(g/L)			Mass Fraction/wt%
	Na_2_SO_4_	NaCl	NaHCO_3_	
Composite salt solution	13.36	7.46	14.38	3.4

## Data Availability

Data are contained within the article.
